# West Nile Virus and Other Nationally Notifiable Arboviral Diseases — United States, 2023

**DOI:** 10.15585/mmwr.mm7421a1

**Published:** 2025-06-12

**Authors:** Hannah Padda, Daniel Jacobs, Carolyn V. Gould, Rebekah Sutter, Jennifer Lehman, J. Erin Staples, Shelby Lyons

**Affiliations:** ^1^Division of Vector-Borne Diseases, National Center for Emerging and Zoonotic Infectious Diseases, CDC; ^2^Epidemic Intelligence Service, CDC; ^3^Alaka`ina Foundation, Orlando, Florida.

**Summary**

**What is already known about this topic?**

U.S. arboviral infections occur primarily through bites from infected mosquitoes or ticks. West Nile virus (WNV) is the leading cause of arboviral disease, which typically manifests as acute febrile or neurologic illness.

**What is added by this report?**

In 2023, arboviral disease cases were reported from all contiguous U.S. states; most occurred during April–December. WNV cases were most common, followed by Powassan virus disease cases, which increased from the previous record high in 2022. Three WNV disease cases were reported among patients infected though organ transplantation from two donors. La Crosse virus was the most common cause of arboviral disease among children, with most cases classified as neuroinvasive.

**What are the implications for public health practice?**

Clinicians should consider arboviral testing for patients with acute febrile or neurologic illnesses when ticks and mosquitoes are active. Timely surveillance is needed to identify areas that pose a risk for arboviral infection. Personal prevention, vector control, and blood and organ donor screening for WNV are essential to reduce arboviral disease morbidity and mortality.

## Abstract

In the United States, arthropodborne viruses (arboviruses) are primarily transmitted by infected mosquitoes or ticks. Most infections are asymptomatic; symptomatic infections range from mild febrile illness to severe neuroinvasive disease. This report summarizes 2023 data for nationally notifiable domestic arboviral diseases. Forty-eight states and the District of Columbia reported 2,770 human arboviral disease cases, including 2,022 (73%) hospitalizations and 208 (8%) deaths. As in previous years, West Nile virus (WNV) was the most commonly reported domestic arboviral disease in 2023, accounting for 2,628 (95%) of all reported cases. A majority (91%) of case onsets occurred during July–September. Three WNV disease cases among patients infected though organ transplantation from two donors were reported in 2023. Powassan virus disease case reports were the second most common (n = 49), having increased from the previous record high in 2022, with onsets evenly distributed during April–December. La Crosse virus was the most common cause of arboviral disease among children, with most cases classified as neuroinvasive. Variations in annual arboviral disease incidence, distribution, and seasonal temporality highlight the importance of high-quality and timely surveillance. Clinicians should consider arboviral testing in patients with acute febrile or neurologic illness when mosquitoes and ticks are active and report positive test results to their health department. Reducing arboviral disease morbidity and mortality relies on population use of personal protective measures (e.g., insect repellent and protective clothing), implementing vector control efforts, and screening blood and organ donors for WNV.

## Introduction

Arthropodborne viruses (arboviruses) are maintained in transmission cycles between arthropods and vertebrate hosts, including humans and other animals (*1*). In the United States, humans primarily become infected through the bites of infected mosquitoes or ticks; although rare, transmission can occur through blood transfusion or organ transplantation (*2*). The leading cause of arboviral disease in the United States is West Nile virus (WNV), which causes an average of 2,000 disease cases annually, including 1,200 life-threatening neurological illnesses and approximately 120 deaths (*3*). Other domestic arboviruses can cause sporadic cases and occasional outbreaks (*3*). Most infections are asymptomatic. Symptomatic infections can range from a mild febrile illness to severe neuroinvasive disease (*1*). Because no prophylactic agents or specific treatments are currently available for domestic arboviral infections, prevention is essential to reduce disease morbidity and mortality. This report includes an analysis of data for the six nationally notifiable domestic arboviral diseases (West Nile, Powassan, La Crosse, Jamestown Canyon, St. Louis encephalitis, and eastern equine encephalitis virus diseases), which typically have similar clinical features. This report provides an annual update on arboviral disease case numbers and incidence and increase awareness of the seasonal and geographic occurrence and characteristics of arboviral diseases in the United States (*3*,*4*).

## Methods

### Data Source

State health departments voluntarily report cases of the six nationally notifiable domestic arboviral diseases to CDC through ArboNET, the national arbovirus surveillance system, using standard surveillance case definitions that include clinical and laboratory criteria.* Confirmed and probable cases are included in this report; these cases are further classified as neuroinvasive (those with meningitis, encephalitis, acute flaccid paralysis, or other unspecified neurologic manifestation) or nonneuroinvasive (all other cases).

### Analysis

Features of cases of the six domestic arboviral diseases are described, including incidence, demographic and clinical characteristics, period of illness onset, geographic distribution by location of residence, and outcomes. Incidence was calculated using 2023 midyear population estimates from the U.S. Census Bureau^†^ as denominators. Incidence calculations were limited to neuroinvasive disease, though patterns seen for all cases are similar, because neuroinvasive disease cases are more consistently diagnosed and reported owing to severity of illness (*1*). Patient demographic and other characteristics were compared with previous year case data reported to ArboNET to identify consistencies and differences. This activity was reviewed by CDC, deemed not research, and conducted consistent with applicable federal law and CDC policy.^§^

## Results

### Reported Arboviral Disease Cases

A total of 2,770 confirmed (812; 29%) and probable (1,958; 71%) domestic arboviral disease cases with illness onset in 2023 were reported to CDC (Table 1). Overall, WNV accounted for 2,628 (95%) cases, followed by Powassan (49; 2%), La Crosse (35; 1%), Jamestown Canyon (27; 1%), St. Louis encephalitis (21; 1%), eastern equine encephalitis (seven; <1%), and unspecified California serogroup viruses^¶^ (three; <1%). Cases were reported from 772 (25%) of 3,143 U.S. counties and all 48 states in the contiguous United States and the District of Columbia (DC). More than one half (57%) of reported cases occurred in patients aged ≥60 years (except for La Crosse virus disease, incidence of which was highest in children), and 63% of all cases occurred in males.

**TABLE 1:** Confirmed* and probable^†^ cases of nationally notifiable arboviral disease, by virus type and selected patient characteristics (N = 2,770)^§^ — United States, 2023

Characteristic	Virus type, no. (%)^¶^ of cases					
	West Nile n = 2,628	Powassan n = 49	La Crosse n = 35	Jamestown Canyon n = 27	St. Louis encephalitis n = 21	Eastern equine encephalitis n = 7
**Age group, yrs**						
<18	40 (2)	4 (8)	32 (91)	3 (11)	0 (—)	2 (29)
18–59	1,072 (41)	11 (22)	1 (3)	10 (37)	7 (33)	1 (14)
≥60	1,516 (58)	34 (69)	2 (6)	14 (52)	14 (67)	4 (57)
Median age (IQR)	63 (49–71)	68 (58–72)	7 (4–11)	60 (40–73)	62 (54–73)	64 (7–72)
**Sex**						
Female	965 (37)	17 (35)	14 (40)	7 (26)	7 (33)	1 (14)
Male	1,663 (63)	32 (65)	21 (60)	20 (74)	14 (67)	6 (86)
**Quarter of illness onset**						
Jan–Mar	7 (<1)	2 (4)	0 (—)	0 (—)	0 (—)	0 (—)
Apr–Jun	73 (3)	16 (33)	0 (—)	10 (37)	1 (5)	2 (29)
Jul–Sep	2,389 (91)	16 (33)	27 (77)	13 (48)	17 (81)	5 (71)
Oct–Dec	155 (6)	15 (31)	8 (23)	4 (15)	3 (14)	0 (—)
Unknown	4 (<1)	0 (—)	0 (—)	0 (—)	0 (—)	0 (—)
**Clinical syndrome**						
Nonneuroinvasive	839 (32)	2 (4)	1 (3)	7 (26)	7 (33)	0 (—)
Neuroinvasive**	1,789 (68)	47 (96)	34 (97)	20 (74)	14 (67)	7 (100)
Encephalitis	1,107 (62)	34 (72)	31 (91)	17 (85)	5 (36)	5 (71)
Meningitis	464 (26)	2 (4)	3 (9)	3 (15)	2 (14)	0 (—)
AFP^††,§§,¶¶^	70 (4)	7 (15)	0 (—)	0 (—)	0 (—)	1 (14)
Unspecified	148 (8)	4 (9)	0 (—)	0 (—)	7 (50)	1 (14)
**Outcome**						
Hospitalization	1,891 (72)	44 (90)	35 (100)	25 (93)	17 (81)	7 (100)
Death	194 (7)	8 (16)	0 (—)	3 (11)	2 (10)	1 (14)

**Abbreviations**: AFP = acute flaccid paralysis; CSF = cerebrospinal fluid; Ig = immunoglobulin.

* A confirmed case meets clinical criteria for arboviral disease and at least one of the following laboratory criteria: 1) isolation of virus from, or demonstration of specific viral antigen or nucleic acid in, tissue, blood, CSF, or other body fluid; 2) fourfold or higher change in virus-specific quantitative antibody titers in paired sera; 3) virus-specific IgM antibodies in serum with confirmatory virus-specific neutralizing antibodies in the same or a later specimen; or 4) virus-specific IgM antibodies in CSF and a negative test result for other IgM antibodies in CSF for arboviruses endemic in the region where exposure occurred.

^†^ A probable case meets clinical criteria for arboviral infection and virus-specific IgM antibodies in CSF or serum but without other testing.

^§^ Three unspecified California serogroup virus cases were also reported but are not included in the table.

^¶^ Percentages might not sum to 100 because of rounding.

** Percentages of cases of encephalitis, meningitis, AFP, and unspecified neurologic signs or symptoms are percentages of neuroinvasive cases.

^††^ Among the 70 West Nile virus disease cases with AFP, 25 (36%) also had encephalitis or meningitis.

^§§^ Among the seven Powassan virus disease cases with AFP, six also had encephalitis.

^¶¶^ The one eastern equine encephalitis virus disease case with AFP also had encephalitis.

### West Nile Virus Disease

The 2,628 WNV disease cases identified in 2023 were reported from 692 (22%) counties in 46 states and DC. Similar to previous years, a majority of patients (91%) had illness onset during July–September. The median patient age was 63 years (IQR = 49–71 years), 63% were male, and 1,789 (68%) had neuroinvasive disease. A total of 1,891 (72%) patients were hospitalized, including 1,665 (88%) who had neuroinvasive disease. Overall, 194 (7%) patients with reported WNV died, including one of three patients who were infected through organ transplants received from two asymptomatic donors. The median age of all patients who died was 73 years (IQR = 66–82 years); 190 (98%) had neuroinvasive disease.

The national incidence of WNV neuroinvasive disease was 0.53 cases per 100,000 population (Table 2). The highest WNV neuroinvasive disease incidences were in Colorado (5.38 per 100,000), South Dakota (5.00), and Nebraska (4.60) (Figure); these states also had the highest incidence of total cases.** The largest numbers of WNV neuroinvasive disease cases were reported from California (334), Colorado (316), and Texas (122), accounting for 43% of neuroinvasive disease cases nationwide. WNV neuroinvasive disease incidence increased with age from 0.02 per 100,000 among persons aged <10 years to 1.56 per 100,000 among those aged ≥70 years. Incidence of WNV neuroinvasive disease was higher among males (0.68 per 100,000) than among females (0.38).

**TABLE 2:** Confirmed* and probable^†^ cases and incidence^§^ of nationally notifiable arboviral neuroinvasive disease, by virus type and U.S. Census Bureau division and jurisdiction — United States, 2023

U.S. Census Bureau division/jurisdiction	No. (incidence) of neuroinvasive disease cases, by virus type*					
	West Nile	Powassan	La Crosse	Jamestown Canyon	St. Louis encephalitis	Eastern equine encephalitis
**United States**	**1,789 (0.53)**	**47 (0.01)**	**34 (0.01)**	**20 (0.01)**	**14 (<0.01)**	**7 (<0.01)**
**New England**	**11 (0.07)**	**28 (0.18)**	**—^¶^**	**—**	**—**	**—**
Connecticut	4 (0.11)	5 (0.14)	—	—	—	—
Maine	—	7 (0.50)	—	—	—	—
Massachusetts	5 (0.07)	10 (0.14)	—	—	—	—
New Hampshire	1 (0.07)	4 (0.29)	—	—	—	—
Rhode Island	1 (0.09)	1 (0.09)	—	—	—	—
Vermont	—	1 (0.15)	—	—	—	—
**Middle Atlantic**	**82 (0.20)**	**8 (0.02)**	**—**	**2 (<0.01)**	**—**	**—**
New Jersey	11 (0.12)	—	—	1 (0.01)	—	—
New York	53 (0.27)	6 (0.03)	—	1 (0.01)	—	—
Pennsylvania	18 (0.14)	2 (0.02)	—	—	—	—
**East North Central**	**175 (0.37)**	**2 (<0.01)**	**14 (0.03)**	**16 (0.03)**	**—**	**—**
Illinois	104 (0.83)	—	—	—	—	—
Indiana	9 (0.13)	—	1 (0.01)	—	—	—
Michigan	22 (0.22)	—	—	5 (0.05)	—	—
Ohio	16 (0.14)	—	12 (0.10)	—	—	—
Wisconsin	24 (0.41)	2 (0.03)	1 (0.02)	11 (0.19)	—	—
**West North Central**	**305 (1.40)**	**8 (0.04)**	**2 (0.01)**	**2 (0.01)**	**—**	**—**
Iowa	12 (0.37)	—	1 (0.03)	—	—	—
Kansas	46 (1.56)	—	—	—	—	—
Minnesota	44 (0.77)	8 (0.14)	1 (0.02)	2 (0.03)	—	—
Missouri	33 (0.53)	—	—	—	—	—
Nebraska	91 (4.60)	—	—	—	—	—
North Dakota	33 (4.21)	—	—	—	—	—
South Dakota	46 (5.00)	—	—	—	—	—
**South Atlantic**	**75 (0.11)**	**1 (<0.01)**	**13 (0.02)**	**—**	**1 (<0.01)**	**3 (<0.01)**
Delaware	4 (0.39)	—	—	—	—	—
District of Columbia	1 (0.15)		—	—	—	—
Florida	9 (0.04)	—	—	—	—	2 (0.01)
Georgia	17 (0.15)	—	—	—	—	1 (0.01)
Maryland	9 (0.15)	1 (0.02)	—	—	—	—
North Carolina	15 (0.14)	—	5 (0.05)	—	—	—
South Carolina	9 (0.17)	—	1 (0.02)	—	1 (0.02)	—
Virginia	9 (0.10)	—	2 (0.02)	—	—	—
West Virginia	2 (0.11)	—	5 (0.28)	—	—	—
**East South Central**	**75 (0.38)**	**—**	**5 (0.03)**	**—**	**—**	**3 (0.02)**
Alabama	25 (0.49)	—	—	—	—	3 (0.06)
Kentucky	10 (0.22)	—	—	—	—	—
Mississippi	27 (0.92)	—	—	—	—	—
Tennessee	13 (0.18)	—	5 (0.07)	—	—	—
**West South Central**	**216 (0.51)**	**—**	**—**	**—**	**—**	**1 (<0.01)**
Arkansas	7 (0.23)	—	—	—	—	—
Louisiana	46 (1.01)	—	—	—	—	1 (0.02)
Oklahoma	41 (1.01)	—	—	—	—	—
Texas	122 (0.40)	—	—	—	—	—
**Mountain**	**504 (1.96)**	**—**	**—**	**—**	**—**	**—**
Arizona	64 (0.86)	—	—	—	—	—
Colorado	316 (5.38)	—	—	—	—	—
Idaho	27 (1.37)	—	—	—	—	—
Montana	23 (2.03)	—	—	—	—	—
Nevada	2 (0.06)	—	—	—	—	—
New Mexico	54 (2.55)	—	—	—	—	—
Utah	5 (0.15)	—	—	—	—	—
Wyoming	13 (2.23)	—	—	—	—	—
**Pacific**	**346 (0.65)**	**—**	**—**	**—**	**13 (0.02)**	**—**
Alaska	—	—	—	—	—	—
California	334 (0.86)	—	—	—	13 (0.03)	—
Hawaii	—	—	—	—	—	—
Oregon	9 (0.21)	—	—	—	—	—
Washington	3 (0.04)	—	—	—	—	—

**Abbreviations**: CSF = cerebrospinal fluid; Ig = immunoglobulin.

* A confirmed case meets clinical criteria for arboviral disease and at least one of the following laboratory criteria: 1) isolation of virus from, or demonstration of specific viral antigen or nucleic acid in, tissue, blood, CSF, or other body fluid; 2) fourfold or higher change in virus-specific quantitative antibody titers in paired sera; 3) virus-specific IgM antibodies in serum with confirmatory virus-specific neutralizing antibodies in the same or a later specimen; or 4) virus-specific IgM antibodies in CSF and a negative test result for other IgM antibodies in CSF for arboviruses endemic in the region where exposure occurred.

^†^ A probable case meets clinical criteria for arboviral infection and virus-specific IgM antibodies in CSF or serum but without other testing.

^§^ Cases per 100,000 population, based on July 1, 2023, U.S. Census Bureau population estimates.

^¶^ Dashes indicate no reported cases.

**FIGURE:**
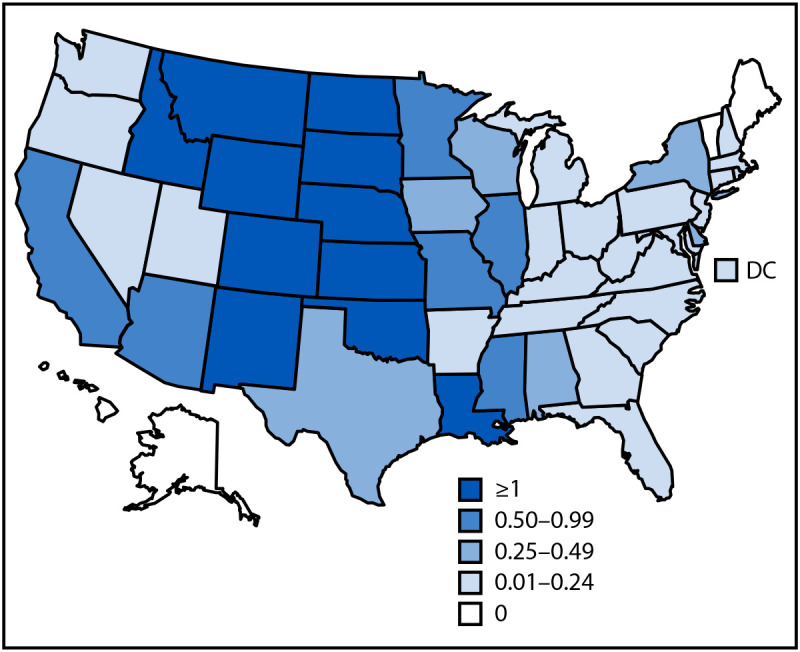
Incidence* of confirmed^†^ and probable^§^ cases of neuroinvasive^¶^ West Nile virus disease, by state — United States, 2023 **Abbreviations:** CSF = cerebrospinal fluid; DC = District of Columbia; Ig = immunoglobulin. * Cases per 100,000 population. ^†^ A confirmed case meets clinical criteria for arboviral disease and at least one of the following laboratory criteria: 1) isolation of virus from, or demonstration of specific viral antigen or nucleic acid in, tissue, blood, CSF, or other body fluid; 2) fourfold or higher change in virus-specific quantitative antibody titers in paired sera; 3) virus-specific IgM antibodies in serum with confirmatory virus-specific neutralizing antibodies in the same or a later specimen; or 4) virus-specific IgM antibodies in CSF and a negative test result for other IgM antibodies in CSF for arboviruses endemic in the region where exposure occurred. ^§^ A probable case meets clinical criteria for arboviral infection and virus-specific IgM antibodies in CSF or serum but without other testing. ^¶^ Cases with meningitis, encephalitis, acute flaccid paralysis, or other unspecified neurologic manifestation.

### Powassan Virus Disease

Forty-nine cases of Powassan virus disease were reported from 11 states, representing the highest number of cases reported to ArboNET since 2004 when Powassan was added as a separate reportable disease condition. Illness onset for 96% of cases occurred during April–December, with cases evenly distributed across this period (Table 1). Two patients, both in New England, experienced illness onset in December. Maryland reported its first Powassan virus disease case in a resident who contracted the virus in Canada.^††^ Median patient age was 68 years (IQR = 58–72 years), and 65% were male. Forty-seven (96%) patients had neuroinvasive disease, 44 (90%) were hospitalized, and eight (16%) died. The median age of patients who died was 71 years (IQR = 67–78 years). States with the highest Powassan virus neuroinvasive disease incidence were Maine (0.50 per 100,000), New Hampshire (0.29), and Vermont (0.15) (Table 2).

### La Crosse Virus Disease

Thirty-five cases of La Crosse virus disease were reported from 10 states. Twenty-seven (77%) patients had illness onset during July–September (Table 1), and, similar to past years, 60% of patients were male, and most patients (91%) were aged <18 years. Thirty-four (97%) patients had neuroinvasive disease. All 35 patients were hospitalized; none died. Ohio reported the highest number of neuroinvasive La Crosse virus disease cases (12; 35%) (Table 2). States with the highest neuroinvasive La Crosse virus disease incidence were West Virginia (0.28 per 100,000), Ohio (0.10), and Tennessee (0.07).

### Jamestown Canyon Virus Disease

Twenty-seven cases of Jamestown Canyon virus disease were reported from seven states. In 85% of cases, illness onset occurred during April–September (Table 1). Median patient age was 60 years, and 74% of patients were male. Twenty (74%) patients had neuroinvasive disease, 25 (93%) were hospitalized, and three (11%) died. The highest incidences of neuroinvasive disease were reported from Wisconsin (0.19 per 100,000), Michigan (0.05), and Minnesota (0.03) (Table 2).

### St. Louis Encephalitis Virus Disease

Twenty-one cases of St. Louis encephalitis virus disease were reported from three states, including the first-ever human disease case reported from South Carolina. Illness onset occurred during July–September in 81% of cases (Table 1). Median patient age was 62 years and 67% were male. Fourteen (67%) patients had neuroinvasive disease, 17 (81%) were hospitalized, and two (10%) died. The highest incidence of St. Louis encephalitis virus neuroinvasive disease was reported in California (0.03 per 100,000) (Table 2).

### Eastern Equine Encephalitis Virus Disease

Seven cases of eastern equine encephalitis virus disease were reported from four states in 2023, which is the same as the median number of cases reported during 2003–2022. Illness onset in five of the seven cases occurred during July–September; two cases occurred during April–June (Table 1). Median patient age was 64 years, and six of the seven patients were male. All seven patients had neuroinvasive disease and were hospitalized; one patient died.

## Discussion

WNV was reported most commonly, and La Crosse virus was the most common cause of neuroinvasive arboviral disease in children among the six notifiable domestic arboviral diseases reported during 2023. Whereas the 2,770 arboviral disease cases reported in 2023 represented more than a doubling of the 1,247 cases reported in 2022 (*3*), this number was lower than the 3,035 cases reported in 2021, when a large focal WNV outbreak in Maricopa County, Arizona accounted for approximately one half of the total cases reported (*4*,*5*). The increase in reported arboviral disease cases from 2022 to 2023 was largely driven by a 132% increase in reported WNV disease cases (from 1,132 in 2022 to 2,628 in 2023), although the number of Powassan virus disease cases reported to ArboNET was the highest since reporting began in 2004, and the number of St. Louis encephalitis virus disease cases was the third highest in 2 decades (*3*).^§§^

Most domestic arboviral disease cases occur during July–September; however, Powassan virus disease cases were more evenly distributed during April–December, and Jamestown Canyon virus transmission began earlier in the spring compared with other mosquitoborne diseases. Differences in seasonality of arbovirus transmission partly reflect differences in the ecology and vectors transmitting these viruses. For example, ticks that can transmit Powassan virus are more active in cooler temperatures than are mosquitoes, and Jamestown Canyon virus is transmitted by a wide variety of mosquitoes (*6*). Changes in weather that support expansions in vector populations or animal hosts also likely affect disease occurrence (*5*).

Arboviral diseases continue to cause substantial morbidity in the United States. Over 7 of the past 10 years (excluding 2019, 2020, and 2022, during which the COVID-19 pandemic likely affected reporting), reported WNV human disease cases have consistently exceeded 2,000 cases per year (*3*,*4*,*7*). Although WNV human disease cases have previously only been associated with lineage 1 and 2 strains of the virus, in 2023, a patient in the United States was found to be coinfected with lineage 1b (the common U.S. strain) and lineage 3, which had only been found before in mosquitoes in central Europe (*8*). The prevalence of lineage 3 in the United States and its significance in human disease and transmission is unknown (*8*). Surveillance for human and animal disease cases and infected vectors are necessary to understand more about this novel lineage. Surveillance is important to lowering arboviral disease incidence by prompting timely prevention messaging and vector control activities.

Since the last WNV transplant transmission cluster was reported in 2018, four additional clusters were identified in 2021, 2022, and 2023 involving eight infected recipients with encephalitis and three deaths (*2*,*3*). These reports underscore the ongoing risk for transmission through solid organ transplantation associated with high morbidity and mortality and the need to potentially screen organ donors for the presence of viral RNA during periods of elevated WNV risk (*2*,*3*).

### Limitations

The findings in this report are subject to at least two limitations. First, because ArboNET does not require inclusion of clinical signs and symptoms or diagnostic laboratory test results in case reports,^¶¶^ cases might be misclassified. Second, ArboNET is a passive surveillance system that relies on patients seeking health care, appropriate testing by clinicians, and reporting of arboviral disease cases; thus, prevalence is likely underestimated. Previous studies estimated that 30–70 nonneuroinvasive disease cases occur for every reported case of WNV neuroinvasive disease (*1*). Based on the 1,789 neuroinvasive disease cases reported in 2023, an estimated 53,670–125,230 nonneuroinvasive disease cases likely occurred; however, only 839 (0.7%–1.6% of the estimated total) were reported.

### Implications for Public Health Practice

Understanding the epidemiology, seasonality, and geographic distribution of domestic arboviruses is important for clinical recognition and guides community messaging efforts and vector control activities. Arboviral disease testing should be considered by clinicians for patients with an acute febrile or neurologic illness, including recipients of organ transplants or blood transfusions, particularly during times when ticks and mosquitoes are active. Testing for the specific causative agent should take into consideration the timing of illness onset and location of exposures. Although most testing can be done commercially, testing for less common viruses and confirmatory testing is often done at public health laboratories. WNV and Powassan virus testing are not typically included in commercial arboviral panels and should be ordered specifically if needed. For Powassan virus disease, the risk for infection is highest from spring to late fall in the Northeast and upper Midwest; thus, messaging (e.g., media and newsletters) to increase patient and provider awareness of the prolonged risk for infection should be considered. Because no prophylactic agents (e.g., human vaccines) or specific treatments (e.g., antiviral medications) are currently available to prevent or treat domestic arboviral infections, management is supportive, and prevention and control rely on personal protective measures (e.g., using insect repellent registered by the Environmental Protection Agency and wearing protective clothing),*** vector control efforts both at household and community levels,^†††^^,§§§^ and WNV blood and organ donor screening to minimize transfusion- and transplant-associated transmission.^¶¶¶^

## Footnotes

*
CDC | National Notifiable Diseases Surveillance System (NNDSS) | Arboviral Diseases, Neuroinvasive and Non-neuroinvasive 2015 Case Definition

†
United States Census Bureau | State Population Totals and Components of Change: 2020–2024

§ 45 C.F.R. part 46, 21 C.F.R. part 56; 42 U.S.C. Sect. 241(d); 5 U.S.C. Sect. 552a; 44 U.S.C. Sect. 3501 et seq.

¶ Unspecified California serogroup virus reports belong to the California serogroup (e.g., La Crosse, Jamestown Canyon, Snowshoe hare, and California encephalitis viruses) but the specific virus is unknown or not identified.

**
Data and Maps for West Nile | West Nile Virus | CDC

††
Maryland Department of Health Announces First Travel-Related Case, Death due to Tickborne Illness Powassan

¶¶ CDC | West Nile Virus | Guidelines for West Nile Virus Surveillance and Control

†††
CDC | Ticks | Preventing Tick Bites
